# Improvement of Dynamic Characteristics of Purpose-Built Vehicles Using Semi-Active Suspension System

**DOI:** 10.3390/s24134310

**Published:** 2024-07-02

**Authors:** Minyoung Kim, Chunhwan Lee

**Affiliations:** 1 Department of Future Mobility Convergence, Chonnam National University, Gwangju 61186, Republic of Korea; mykim09366@jnu.ac.kr; 2 Department of Intelligent Mobility, Chonnam National University, Gwangju 61186, Republic of Korea

**Keywords:** purpose-built vehicle, ride comfort, semi-active suspension system, speed bump shape, vibration dose value

## Abstract

The diversification of mobility into services such as smart stores and conference rooms has accelerated the development of purpose-built vehicles (PBVs)—vehicles designed for specific purposes that utilize an extended electric vehicle chassis and autonomous driving technology. Despite the standards on speed bump dimensions stipulated by the National Land Transportation Act of the Republic of Korea, real-world speed bumps feature varying widths and heights that deviate from these standards. In this study, a velocity equation was derived via regression analysis to achieve the desired dynamic characteristics for a PBV passing over speed bumps with varying shapes through two types of semi-active suspension control: proportional–integral–differential (PID) and linear–quadratic–regulator (LQR). For a cargo-transport PBV, the PID and LQR controllers increased the velocity by 23.74% and 50.74%, respectively, under different speed bump widths and by 19.44% and 38.31%, respectively, under different speed bump heights. Moreover, an analysis of the vibration dose value (*VDV*), an indicator of ride comfort, revealed that the *VDVs* calculated using the velocity equation were within an acceptable error range of 10% above the target *VDV*. These findings provide insights into the speed control required for different types of autonomous PBVs to ensure ride comfort, as well as minimize the driving duration, depending on the specific purpose of the vehicle.

## 1. Introduction

In recent years, progress in the development of electric vehicles and autonomous driving technologies has amplified interest in unmanned autonomous vehicles that integrate these technologies. With regard to mobility, focus is shifting from traditional transportation methods to the optimal utilization of space in vehicles. This transition is exemplified by the development of purpose-built vehicles (PBVs), which utilize electric vehicle batteries and have a long wheelbase. Expanding upon this concept, mobility as a service—an industry that offers a spectrum of services in the form of smart stores and conference rooms, in addition to passenger and cargo transportation—is gaining traction. The fact that PBVs offer features such as beds and smart stores emphasizes the importance of their mechanical stability. However, research on the safeguarding of passengers or payloads in PBVs during the autonomous driving process is insufficient [1]. The stability of PBVs is a key factor that influences user trust in unmanned autonomous vehicles [2]. The establishment and maintenance of a stable driving platform are pivotal factors for ensuring that PBVs achieve initial market penetration and subsequent expansion in the realm of autonomous vehicles. Addressing these concerns is crucial for fostering user confidence and promoting the widespread adoption of PBVs in the evolving landscape of autonomous mobility.

To ensure dynamic stability and optimal ride comfort, the driving method and design variables of PBVs must be tailored to their intended use. However, mass customization of individual PBVs is impractical for automotive companies. Therefore, research should focus on achieving PBV stability through purpose-specific control logic on standardized PBV platforms.

Speed bumps are commonly installed on road surfaces to limit vehicle velocity. To minimize impact, autonomous vehicles typically decelerate at a constant rate when they detect speed bumps. However, variations in speed bump dimensions, particularly in areas where nonstandard bumps are prevalent, such as apartment complexes and university campuses, present complex challenges. Deviations from standard specifications can result in significantly different levels of displacement and vertical acceleration, potentially leading to fractures and injuries for passengers [3,4]. In fact, domestic speed bumps are known to range from 2.1 to 7.6 m in width and from 0.04 to 0.18 m in height [5]. Moreover, a change in height affects ride comfort more than a change in width does; specifically, when the height is increased by 5 cm, the ride comfort is reduced by about 36% for a given vehicle velocity [6].

Given the possibility of PBVs operating on private land and public roads, passenger or payload stability must be ensured by accounting for speed bumps of various dimensions. Therefore, this study focuses on ensuring stability by adjusting the PBVs’ speeds according to the specific dimensions of a speed bump, rather than maintaining the same speed for all types of speed bumps.

To ensure driving stability, the use of semi-active suspension control technology, which facilitates improved ride comfort, has been proposed. The present study focuses on achieving purpose-specific ride comfort in PBVs by employing semi-active suspension control. Numerous studies have attempted to ensure the comfort and stability of vehicle passengers, focusing on mitigating impacts associated with acceleration and vibration during driving.

Drawing from the ISO2631-1 standard [7], one study explored speed control logic to ensure passenger comfort by considering the road-surface roughness [1]. Another study analyzed the vibration caused by speed bumps on road surfaces [8], as well as the dynamic characteristics of speed bumps according to their shapes [9]. Additional research investigated the effects of vertical vibration exposure on passengers, examining factors such as vibration amplitude and frequency [10], and compared subjective and objective indicators of ride comfort [11]. Moreover, dynamic characteristics during speed bump traversal at varying vehicle speeds have been analyzed, leading to the derivation of Pareto-optimum speeds [12]. Researchers have also analyzed ride comfort in a tractor under various road-surface conditions [13] and have studied harmful vibration exposure levels by measuring whole-body vibration under different speed-bump shapes [14].

Research on speed bump detection is also being actively conducted. Recently, a technology with more than 99% accuracy has been reported [15]. Research on speed bump detection is also being actively conducted. Recently, a technology with more than 99% accuracy has been reported [15].

Furthermore, with regard to vehicle modeling, studies have extended beyond the traditional half-car model to examine health risks and ride comfort, optimizing variables for scenarios such as a person lying in an ambulance bed [16,17,18]. Additionally, some studies have analyzed passenger ride comfort based on seat location via vehicle seat modeling [19,20].

To mitigate impacts from road surface-induced vibration and acceleration, studies have explored suspension control methods, in addition to speed control or vehicle design. This includes investigations into ride comfort enhancement using model predictive control [21,22], hybrid fuzzy control [23], and proportional–integral–differential (PID) control, as well as comparisons of dynamic characteristics achieved with various controllers, such as linear–quadratic–regulator (LQR) and fuzzy and and neural network-based controllers [24,25]. In addition, some studies have recently used semi-active suspension control based on reinforcement learning [26].

**Figure 1:**
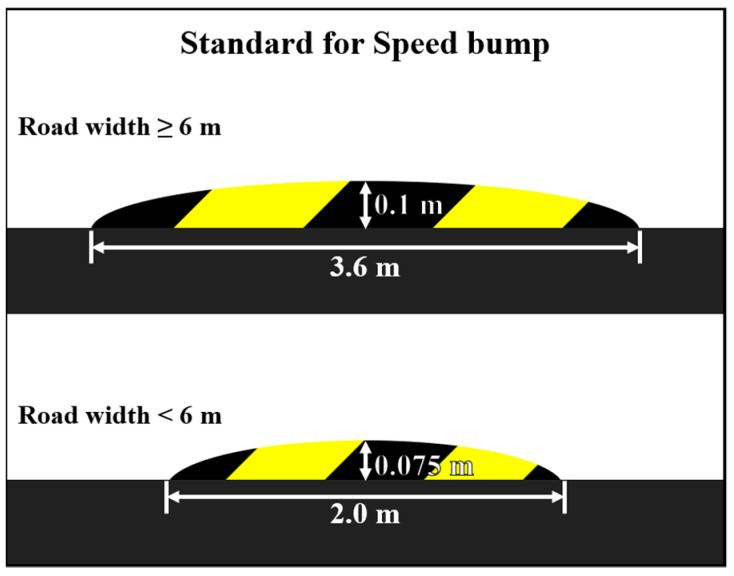
Standard for speed bump dimensions in the Republic of Korea.

The remainder of this paper is structured as follows: Section 2 explains the objective standards for ride comfort and purpose-specific modeling techniques for PBVs. Section 3 describes semi-active suspension control methods and speed bump traversal strategies to secure ride comfort. Section 4 presents the experimental results and their analysis. Finally, Section 5 discusses the conclusions and implications. See Figure 1.

## 2. Standard for Ride Comfort and Modeling of PBV

### 2.1. Standards for Ride Comfort

Although prior studies have compared the comfort and stability of passengers or payloads in a vehicle based on objective and subjective indicators [11], the ISO2631-1 [7] standard, published by the International Organization for Standardization (ISO), was selected in this study to objectively evaluate the comfort and stability of passengers or payloads in a PBV. ISO2631-1 considers health problems, ride comfort, motion sickness, and other factors associated with whole-body vibration. These factors were examined according to the vibration directions (*x*, *y*, and *z* axes) at each seat position, and quantitative values weighted by frequency were employed to ensure a comprehensive evaluation. The important indicators for assessing ride comfort include weighted effective values, such as the weighted root-mean-square acceleration (WRMS) and vibration dose value (*VDV*). The utilization of the ISO2631-1 standard provides a consistent and systematic approach for objectively standardizing and improving the comfort and stability of passengers or cargo in various PBV configurations.

#### 2.1.1. Weighted Root-Mean-Square Acceleration

WRMS is calculated using Equation (1), which includes a weighting function based on the frequency of the acceleration measured by the vehicle and considers the root-mean-square (RMS) of the vibration exposure time (T). Additionally, a correction factor value (Table 1) is applied based on the direction of vibration. The total WRMS, considering the *x*, *y*, and *z* axes, is then calculated using Equation (2).

(1) $$a_{w}={\left[\frac{1}{T}\int_{0}^{T}a_{w}^{2}\left(t\right)dt\right]}^{\frac{1}{2}}$$

(2) $$a_{v}={\left(k_{x}^{2}a_{wx}^{2}+k_{y}^{2}a_{wy}^{2}+k_{z}^{2}a_{wz}^{2}\right)}^{\frac{1}{2}}$$

WRMS serves as a metric for evaluating the effects of prolonged driving, continuous exposure to vibration, and road-surface conditions; thus, it addresses the limitation of acceleration, which does not allow the vibration exposure time to be considered. To understand the relationship between WRMS and passenger comfort, ISO 2631-1 classifies ride comfort based on ranges in WRMS, as shown in Table 2. As seen in this classification, some of the WRMS ranges contain overlapping values; this is because clear thresholds cannot be defined, given that ride comfort is affected by multiple factors, such as noise, temperature, and passenger behavior [7].

In Equation (1), $a_{w}$ represents WRMS, *T* represents the vibration exposure time, and $a_{w}\left(t\right)$ represents the acceleration weighted by a frequency-weighting function. In Equation (2), $a_{wx}$, $a_{wy}$, and $a_{wz}$ represent the WRMS values along the x, y, and z axes, respectively, depending on the direction of vibration. Similarly, $k_{x}$, $k_{y}$, and $k_{z}$ are the respective correction factor values.

**Figure 2:**
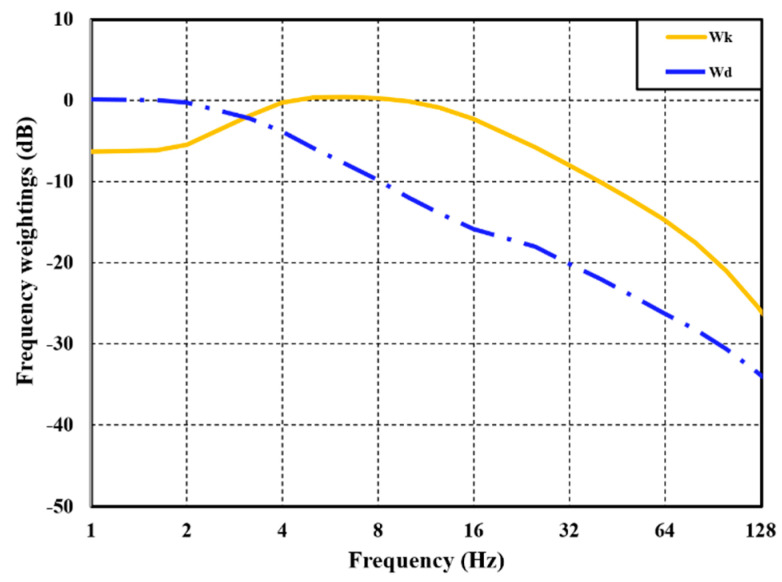
Frequency–weighting curves for vertical (Wk) and horizontal (Wd) directions.

WRMS is known for its sensitivity to vibration exposure time and is, thus, effective for continuous vibration measurement. However, in environments involving high-amplitude vibrations characterized by short exposure times, the assessment of vibration characteristics becomes challenging when considering excessive exposure time. To address this limitation, *VDV* is introduced as an evaluation index. See Figure 2.

#### 2.1.2. Vibration Dose Value

*VDV* represents the cumulative duration of vibration exposure, calculated using the quadruple root of the sum of four squares. This method minimizes the effect of the vibration measurement time and is particularly sensitive to vibration peaks. *VDV* is commonly used for assessing vibrations from sources such as speed bumps and potholes. Similar to WRMS, it is determined using the acceleration weighted by a frequency-weighting function. The total *VDV*, considering all axes (*x*, *y*, and *z*), can be calculated using the quadruple root of the sum of the values obtained for each axis. In (4), i represents the axis (*x*, *y*, and *z*).

(3) $$VDV={\left\{\int_{0}^{T}{\left[a_{w}\left(t\right)\right]}^{4}dt\right\}}^{\frac{1}{4}}$$

(4) $$VDV_{total}={\left(\sum_{i}VDV_{i}^{4}\right)}^{\frac{1}{4}}$$

**Figure 3:**
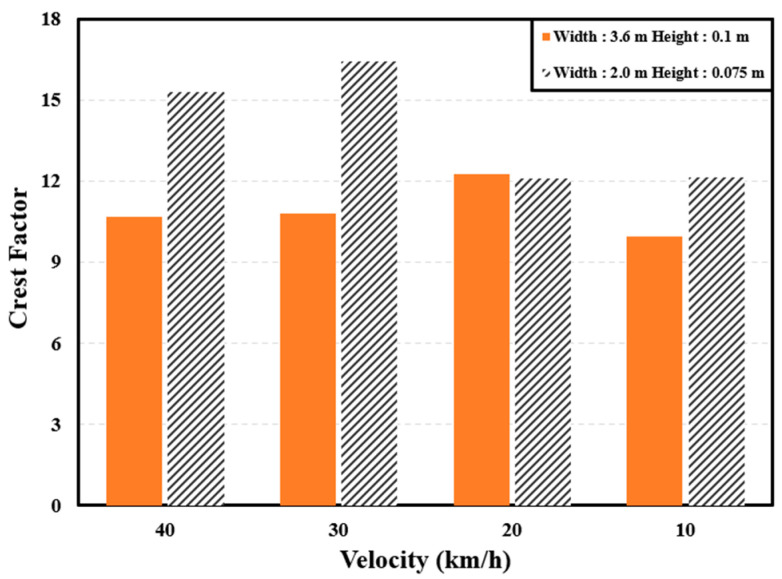
Crest factors when driving over different speed bumps.

To use *VDV* as an evaluation index for vibration, either the exposure time must be short (within 30 s) [8] or the crest factor must be as suggested in ISO2631-1 [7]. ISO2631-1 defines the crest factor as the ratio of the maximum instantaneous peak value of the frequency-weighted acceleration to the WRMS value. ISO recommends using *VDV* when the crest factor is nine or more [7]. Figure 3 presents the crest factors for the accelerations observed when driving over two types of speed bumps at different velocities. As seen in the figure, the crest factor exceeded nine in every case; hence, *VDV* was selected as the ride comfort index in this study.

When evaluating comfort according to *VDV*, an estimated *VDV* (*eVDV*) is used. The comfort levels corresponding to the WRMS values in Table 2 are converted into an *eVDV* based on Equation (5) and used as evaluation indicators.

(5) $$eVDV=1.4a_{w}T^{\frac{1}{4}}$$

### 2.2. Purpose Built Vehicle Modeling

Modeling is essential for mathematically interpreting the behavior of objects, including PBVs. To evaluate the dynamic characteristics of a PBV, various models are employed, such as the full-car, half-car, and quarter-car models, which are categorized according to the degrees of freedom, considering the suspension spring and damper.

**Figure 4:**
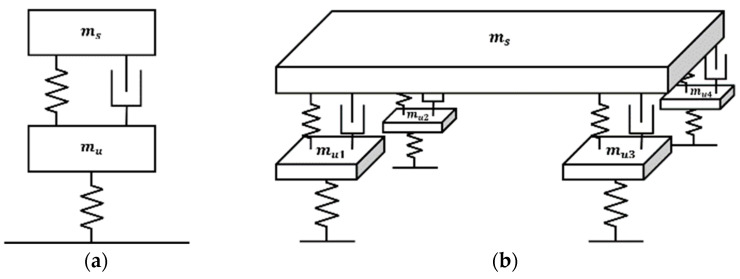
Vehicle models according to degrees of freedom: (**a**) quarter-car model; (**b**) full-car model.

The full-car model, with seven degrees of freedom accounting for all four wheels, can capture diverse vehicle behaviors such as pitch, roll, and heave. However, it also suffers from modeling complexity. Conversely, the quarter-car model, which considers only one wheel, features simpler formulas and is commonly used in research on active suspension systems. However, it cannot capture behaviors such as pitch or the impact of a long wheelbase when the vehicle encounters speed bumps. The half-car model, accounting for two wheels, offers a balance between accuracy and simplicity. It can consider the effect of a long wheelbase and the pitching behavior when a PBV passes over speed bumps. Accordingly, the half-car model, which offers the ability to control the suspension system, was utilized in this study to examine the dynamic behavior of the PBV. See Figure 4.

#### 2.2.1. Cargo Modeling

**Figure 5:**
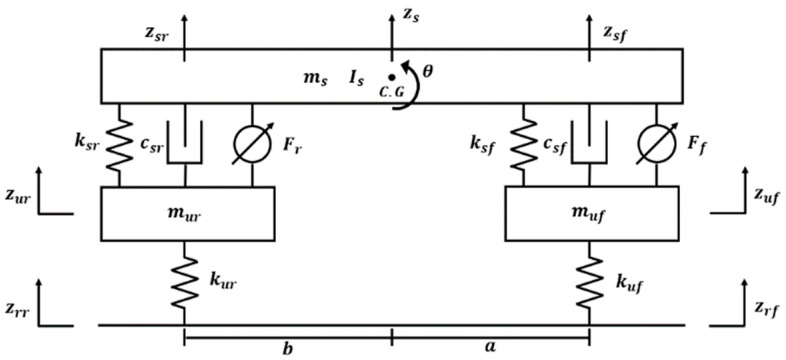
Half-car model with suspension control.

For cargo-transportation PBVs, the payload is typically expected to be loaded on rigid structures, such as floors, shelves, or tables inside the PBV. The half-car model with controllable suspension representing a cargo-carrying configuration is illustrated in Figure 5 [27,28,29]. Based on this model, the differential equations for each degree of freedom are expressed as state-space equations (Equation (A1)). In the PBV model, $m_{s}$ is the sprung mass, and $m_{uf}$ and $m_{ur}$ are the unsprung mass on the front and rear axles, respectively. In addition, springs ($k_{sf},k_{sr}$) and dampers ($c_{sf},c_{sr}$) are present between the sprung and unsprung masses. It is configured by a spring ($k_{uf},k_{ur}$) of the vehicle tire. $I_{s}$ is the inertia of the sprung mass, and $\theta_{s}$ is the pitch. Further, z denotes the displacement of each component.

**Figure 6:**
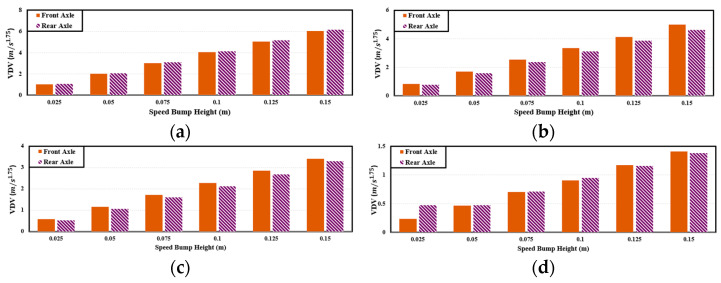
*VDV* comparison between front and rear axles for cargo-carrying PBV: (**a**) 40 km/h; (**b**) 30 km/h; (**c**) 20 km/h; (**d**) 10 km/h; speed bump width: 3.6 m.

In this study, the cargo was assumed to be loaded perpendicular to the wheel axis on the floor inside the PBV. To simplify calculations, the dynamic characteristics were analyzed by comparing the *VDVs* on the front and rear wheels to select the axle with the higher average *VDVs*. Figure 6 illustrates the comparison of the *VDVs* between the front and rear axles, considering a speed-bump width of 3.6 m and a varying PBV velocity. Based on this Figure 6, on average, the *VDVs* are higher on the front axle of the PBV. Accordingly, the dynamic characteristics were analyzed considering only the front axle. It was assumed that if appropriate dynamic behavior was achieved on the front axle, a similar behavior would also be observed on the rear axle.

#### 2.2.2. Passenger Seat Modeling

PBVs are anticipated to be widely used in passenger transportation, a fundamental requirement for vehicles. For a PBV to serve as a meeting space, the seats are expected to be symmetrically arranged, facing each other. This configuration is represented in the half-car model, considering both the front and rear seats [19,20]. In the passenger seat model, $m_{Pf}$ and $m_{Pr}$ are the masses of the seats. Springs ($k_{Pf},k_{Pr}$) and dampers ($c_{Pf},c_{Pr}$) are present between the sprung mass and the seats. It is configured by a spring ($k_{uf},k_{ur}$) of the PBV tire. Further, z represents the displacement of each component. Based on this model, the differential equations for each degree of freedom are expressed as state-space equations (Equation (A2)). 

**Figure 7:**
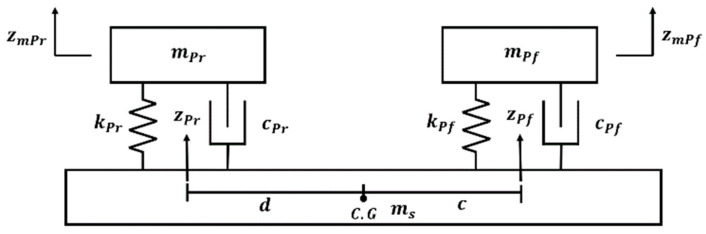
Passenger seat model.

**Figure 8:**
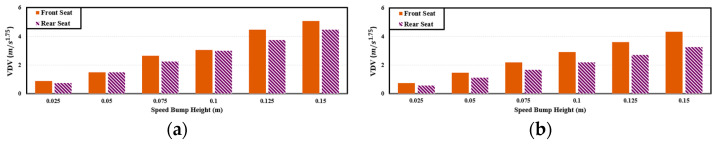

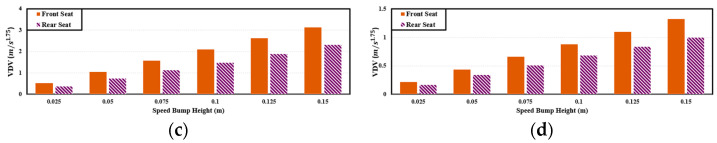
*VDV* comparison between front and rear axles for passenger-transport PBV: (**a**) 40 km/h; (**b**) 30 km/h; (**c**) 20 km/h; (**d**) 10 km/h; speed bump width: 3.6 m.

Similar to cargo-carrying PBVs, PBVs intended for passenger transportation must be assessed for comfort in both front and rear seats. See Figure 7. Figure 8 presents a comparison of *VDV* between the front and rear seats, which can serve as an evaluation metric for suspension and speed control in the future. The assessment is based on the front seats, which exhibit higher *VDVs* on average. 

#### 2.2.3. Bed Modeling

**Figure 9:**
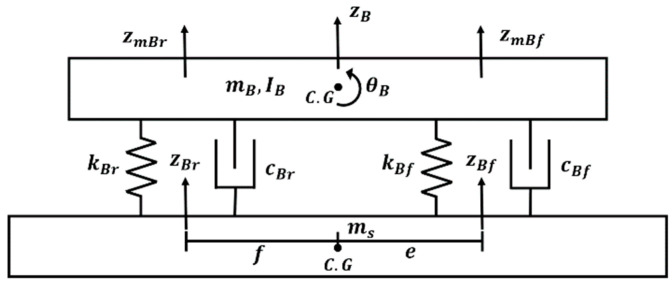
Bed model in PBV.

The future of mobility is envisioned to encompass versatile applications, with PBVs offering sleeping arrangements, as well as medical services, via installed mattresses. Thus, ensuring ride comfort for passengers lying on a bed within a PBV is paramount. To model the bed, it was integrated into the previously constructed half-car model [16,17,18]. Based on this model, the differential equations for each degree of freedom are expressed as state-space equations (Equation (A3) Appendix A). When a person lies on a bed, the analysis must focus on the perception of the ride resulting from the acceleration applied to the head. The head was assumed to be located at a point with a distance e from the center of gravity of the bed. See Figure 9.

#### 2.2.4. Comparison of Dynamic Characteristics between Purpose-Specific Models

The dynamic characteristics of the cargo, passenger, and bed models were analyzed considering a scenario where the PBV passed over a speed bump at a constant velocity of 30 km/h. For an objective assessment, two parameters—acceleration and *VDV*—were examined in accordance with the speed bump specifications outlined by the Ministry of Land, Infrastructure, and Transport of the Republic of Korea.

**Figure 10:**
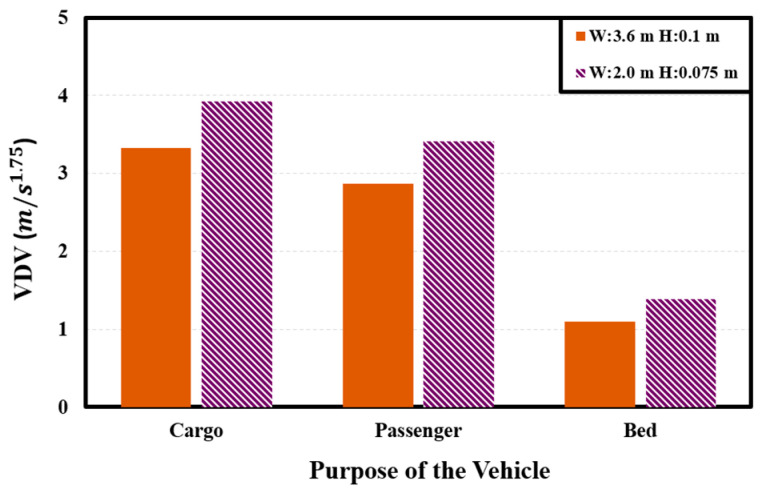
*VDV* observed for different PBV configurations.

Figure 10 depicts the vertical (*z*-axis) acceleration when the PBV passes over the speed bumps. Figure 10 reveals a noticeable trend: the peak-to-peak acceleration and *VDVs* are significant, and the values are the highest for the cargo configuration, followed by the passenger and bed configurations. This observation underscores the necessity of ensuring ride stability by tailoring the PBV modeling process to the specific purpose of the application. See Table 3.

## 3. Suspension Controller Design and Target Velocity Setting Process

### 3.1. Semi-Active Suspension Control

Herein, a suspension control system is proposed to enhance ride comfort and improve handling, particularly over road-surface irregularities such as speed bumps. The primary objectives of this suspension control system are to mitigate the impact perceived by occupants and enhance vehicle handling.

**Figure 11:**
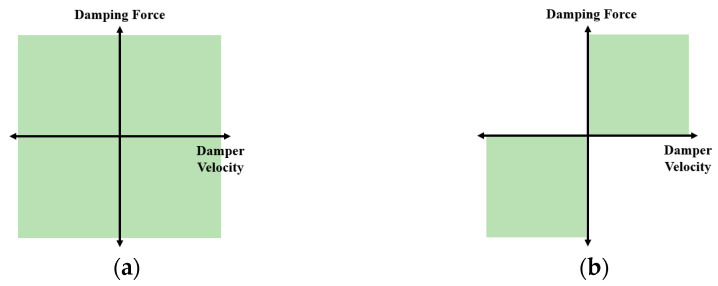
Control areas of active and semi-active suspension systems: (**a**) active suspension control area; (**b**) semi-active suspension control area.

Control methods for suspension systems are typically categorized into passive, semi-active, and active types. Active systems, particularly those incorporating air springs, can adjust the damping force under various driving conditions. However, owing to factors such as cost and durability issues, this technology is predominantly restricted to high-end vehicles. As an alternative, semi-active suspension systems offer a balance between control capability and cost-effectiveness. Although their control range may be more limited than that of active suspension systems, semi-active systems provide a pragmatic solution by adjusting the damping force appropriately during vehicle motion. This approach considers both utilization and economic feasibility and, therefore, represents a viable choice for a wide range of PBVs. See Figure 11.

#### 3.1.1. Proportional–Integral–Differential Control

PID control, implemented as a feedback control loop, mitigates errors by computing the disparity between inputs; this is achieved by feeding back the output to the controller. PID is one of the most widely utilized types of controllers in the industry. The controller inputs proportional, integral, and differential calculated values into the plant according to the error values. In this study, the PID gains were tuned to a value that would reduce the transition time and overshoot.

(6) $$u\left(t\right)=K_{p}e\left(t\right)+K_{i}\int_{0}^{t}e\left(\tau \right)d\tau +K_{d}\frac{de(t)}{dt}$$

(7) $$e\left(t\right)=r\left(t\right)-y(t)$$

#### 3.1.2. Linear-Quadratic-Regulator Control

An LQR controller operates by utilizing full-state feedback based on state-space equations. LQR control, a form of optimal control, involves establishing appropriate weighting factors. In Equation (10), Q and R determine the optimality of LQR control [30]. In general, the Q and R matrices are selected to be diagonal matrices, and the R matrix should be large for small inputs, while the Q matrix should be large for small states. When the Q matrix is fixed, if the R matrix decreases, the transition time and overshoot decrease, but the rise time and steady-state error increase. Conversely, if the R matrix is constant and the Q matrix decreases, the rise time and steady-state error decrease [31]. In this study, to reduce the transition time and overshoot, a larger weight value was assigned to the Q matrix, as seen in Equations (A4) and (A7). In Equations (A4)–(A7), $Q_{f}$ and $R_{f}$ are the values selected for the LQR control applied to the front suspension, while $Q_{r}$ and $R_{r}$ denote the values selected for the rear suspension control. In the state-space Equation (8), the feedback is incorporated by considering the K value representing the state feedback gain Equation (9). The system’s state-corrected weight matrix Q and the input-corrected weight matrix R are selected accordingly, and the optimal gain for minimizing the cost function Equation (10) is calculated using Equations (11) and (12), which are algebraic Riccati equations.

(8) $${\dot{z}}_{H}=A_{H}z_{H}+B_{F}u_{F}$$

(9) $$u_{F}=-Kz_{H}$$

(10) $$J=\int \left(z_{H}^{T}Qz_{H}+u_{F}^{T}Ru_{F}\right)dt$$

(11) $$A_{H}^{T}P+PA_{H}-PB_{F}R^{-1}B_{F}^{T}P+Q=0$$

(12) $$K=R^{-1}B_{F}^{T}P$$

### 3.2. Strategy for Speed Bump Traversal

This study focuses on establishing a correlation between the shapes of speed bumps and their widths and heights via multiple regression analysis. An additional objective is to determine the speed as per user-defined dynamic characteristics to ensure stability when driving over speed bumps [9].

To determine speed variations according to the shape of a speed bump, the speed bump characteristics must be clearly understood. Conventional navigation systems only indicate the presence or absence of speed bumps, without describing their shape. Therefore, the use of a sensor installed in the PBV is proposed to accurately measure the sizes and shapes of speed bumps. In this study, the shape of the speed bump is assumed to be precisely measured by the sensor.

#### 3.2.1. Creation of the Simulation Environment

Using the Carmaker simulation tool (IPG Automotive), speed bumps with specified widths and heights were added at regular intervals on a road surface in a virtual environment. Road profiles were then applied to both the front and rear wheels to simulate the speed and behavior of the PBV. To ensure that the PBV remains stable when crossing a speed bump, it is crucial to analyze its dynamic characteristics at different velocities.

**Figure 12:**
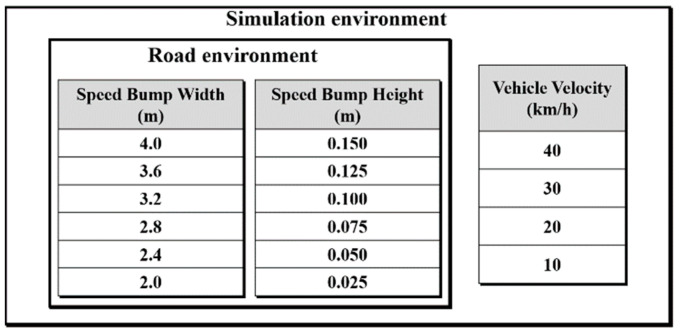
Simulation environment parameters.

**Figure 13:**
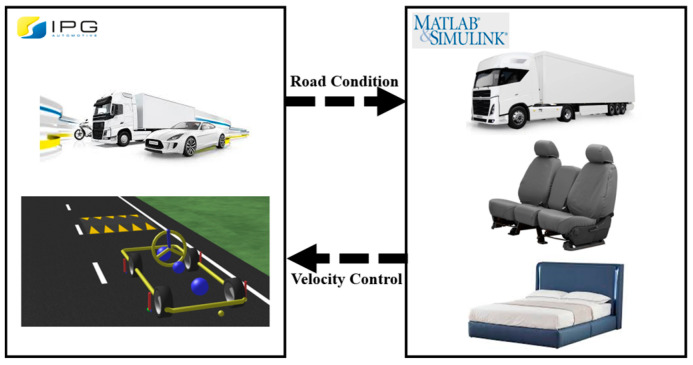
Simulation software interworking diagram.

Considering the speed limit regulations for autonomous PBVs currently under research and development, simulations were conducted in the environment illustrated in Figure 12 The analysis focused on the dynamic characteristics of the PBV when passing over speed bumps at different velocities. See Figure 13.

#### 3.2.2. Correlation between Dynamic Characteristics and Speed Bump Shape

**Figure 14:**
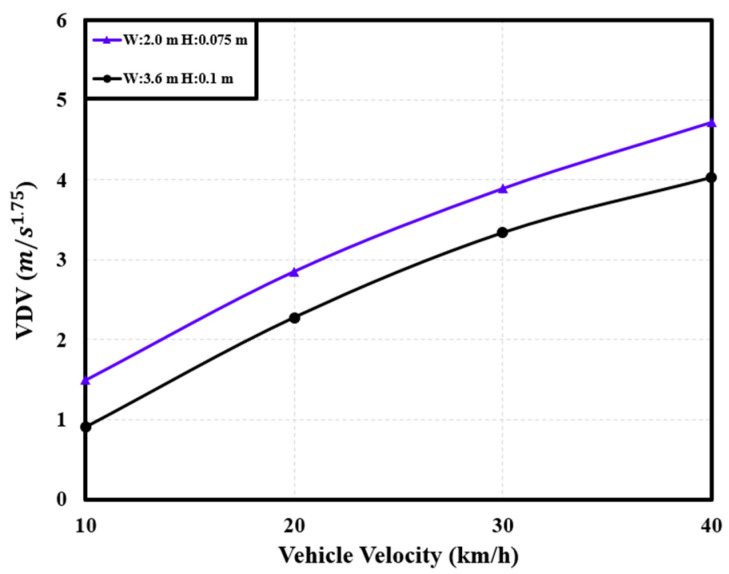
Variations in the *VDV* with PBV velocity.

**Figure 15:**
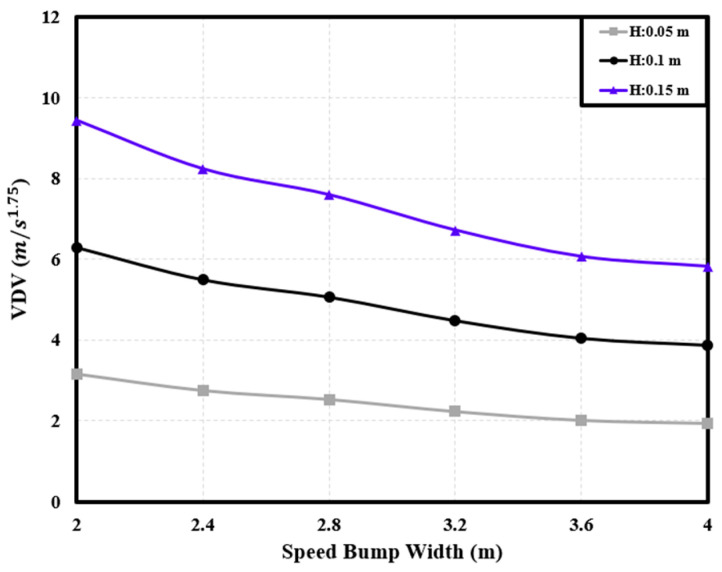
Variations in the *VDV* with speed bump width.

**Figure 16:**
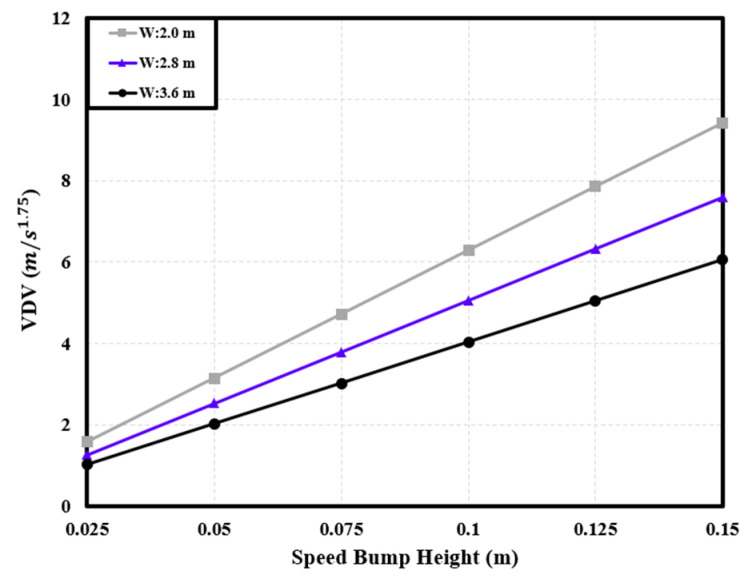
Variations in the *VDV* with speed bump height.

The installation of a speed bump involves considerations of both width and height. When driving over a speed bump manufactured in a nonstandard form, the dynamic characteristics of the PBV can vary depending on the specific dimensions of the speed bump [6]. Hence, considering a uniform velocity (40 km/h), the dynamic characteristics of the cargo-carrying PBV were analyzed for speed bumps with different widths and heights to understand how the PBV responded to variations in these parameters. See Figure 14, Figure 15 and Figure 16.

#### 3.2.3. Velocity Equation via Multiple Regression Analysis

Multiple regression analysis is a statistical method applied when two or more independent variables exist. In this study, multiple regression analysis was utilized to derive regression equations describing the comfort level, considering the width and height of the speed bump and the PBV velocity at the speed bump as independent variables [6]; the dependent variable was the *VDV*. The dynamic characteristics of the PBV can vary depending on the suspension control method, and the velocity at which the PBV passes over the speed bump affects comfort. Therefore, separate regression equations were established for each controller. The analysis was conducted using MATLAB (MathWorks).

Equation (13) represents the relationship between the shape of the speed bump and the *VDV* with respect to the PBV velocity. On this basis, Equation (14) is derived by summarizing the equation for the PBV velocity. By detecting the width and height of the speed bump and substituting the *VDV* into Equation (14), the velocity that ensures the desired comfort level when passing over the speed bump can be determined.

(13) $$VDV_{x}=a_{1}-a_{2}\cdot W_{x}+a_{3}\cdot e^{\left(a_{4}+a_{5}\cdot H_{x}+a_{6}\cdot V_{x}\right)}$$

(14) $$V_{x}=\frac{\left\{\ln\left(VDV_{x}-a_{1}+a_{2}\cdot W_{x}\right)\right\}/a_{3}-a_{4}-a_{5}\cdot H_{x}}{a_{6}}$$

## 4. Experimental Results and Analysis

This section discusses the analysis and verification of the effectiveness of the speed Equation (14) to ensure dynamic stability when the PBV passes over a speed bump.

### 4.1. Velocity Comparison for Suspension Control Using the Velocity Equation

#### 4.1.1. Cargo

**Figure 17:**
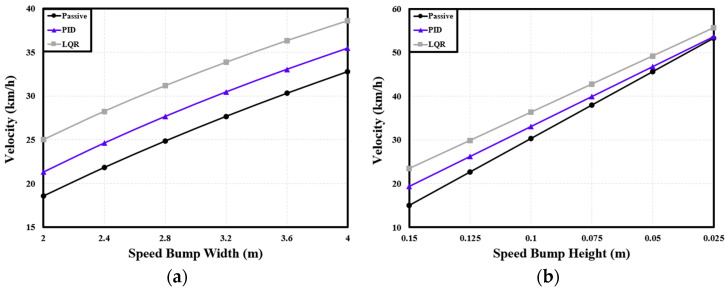
Variations in velocity of cargo PBV with speed bump shapes: (**a**) velocity with speed bump width for a height of 0.1 m; (**b**) velocity with speed bump height for a width of 3.6 m.

Focusing on the front axle, a cargo-transportation experiment was conducted to analyze the variation in speed with the shape of the speed bump. Figure 17a illustrates the velocity variation over speed bumps of different widths and a constant height of 0.1 m. Additionally, Figure 17b displays the velocity variation over speed bumps of different heights and a set width of 3.6 m.

Table 4 lists the differences in velocity achieved with the two suspension control methods under the different speed bump widths. Compared with the passive suspension, the PID controller increased the velocity by an average of 11.01%, while the LQR controller increased the velocity by 24.93% on average. Table 5 lists the velocity differences produced by the two suspension control methods under different speed bump heights. Compared with the passive suspension, the PID and LQR controllers increased the velocity by 10.24% and 22.13%, respectively, on average.

#### 4.1.2. Passenger

Based on the modeling described in Section 2, the behavior of the passenger-transport PBV was examined in terms of its velocity while passing over speed bumps of various shapes. The analysis focused on the dynamic characteristics measured at the front seat, with a road environment similar to that used for the cargo-transportation scenario.

**Figure 18:**
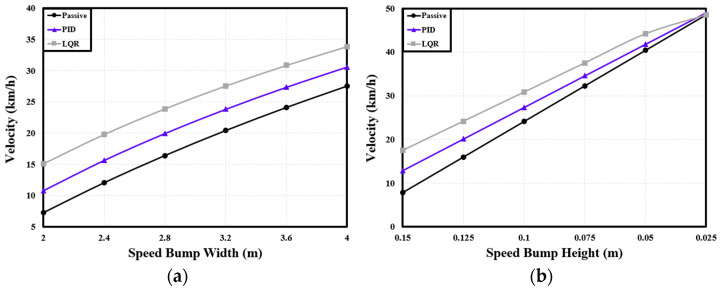
Variations in velocity of passenger PBV with speed bump shapes: (**a**) velocity with speed bump width for a height of 0.1 m; (**b**) velocity with speed bump height for a width of 3.6 m.

Table 6 presents the differences in velocity achieved with the two suspension control methods under different widths of the speed bump. Compared with the passive suspension, the PID controller increased the velocity by an average of 23.74%, while the LQR controller increased the velocity by 50.71% on average. Table 7 presents the velocity differences produced by the two suspension control systems under different speed bump heights. Relative to the passive suspension, the PID and LQR controllers increased the velocity by 19.44% and 38.31%, respectively, on average. See Figure 18.

#### 4.1.3. Bed

As discussed earlier, the inclusion of beds within PBVs is necessary in anticipation of applications involving sleeping arrangements or ambulance services. For such applications, factors such as passenger satisfaction and safety become crucial, along with the necessity of reducing driving time.

Considering the changes in the width of the speed bump, when the PBV passed over speed bumps, the PID-based semi-active suspension system increased the speed by 4.27 km/h on average compared with the passive suspension system, representing a 22.28% increment; with the LQR-based system, the velocity was increased by 7.6 km/h, indicating a 40.59% increment. Considering the changes in the height of the speed bump, the PID controller increased the velocity by an average of 3.45 km/h (20.54%), while the LQR controller increased the velocity by 5.72 km/h (34.21%) on average.

**Figure 19:**
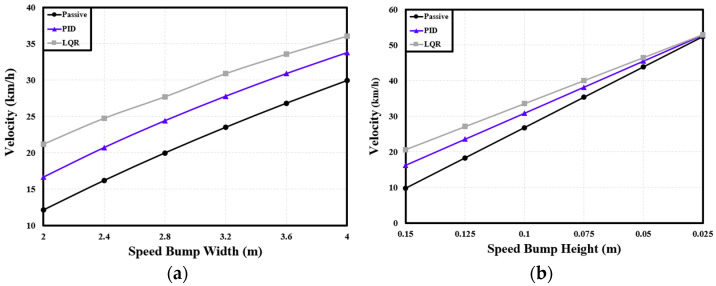
Variations in velocity of bed PBV with speed bump shapes: (**a**) velocity with speed bump width for a height of 0.1 m; (**b**) velocity with speed bump height for a width of 3.6 m.

The findings from the analyses described in this section reveal consistent trends for all three types of PBV with regard to the velocity difference produced by the suspension control system when the PBV passed over speed bumps with different dimensions. However, the velocity difference was noticeably diminished when the height of the speed bump was reduced. This observation suggests that the height of the speed bump has a substantial impact on the velocity of the PBV during speed bump traversal. If the height is extremely low, the velocity difference is reduced as the impact is mitigated. See Table 8 and Table 9 and Figure 19.

### 4.2. Comparison of Acceleration Based on User-Defined Comfort

**Figure 20:**
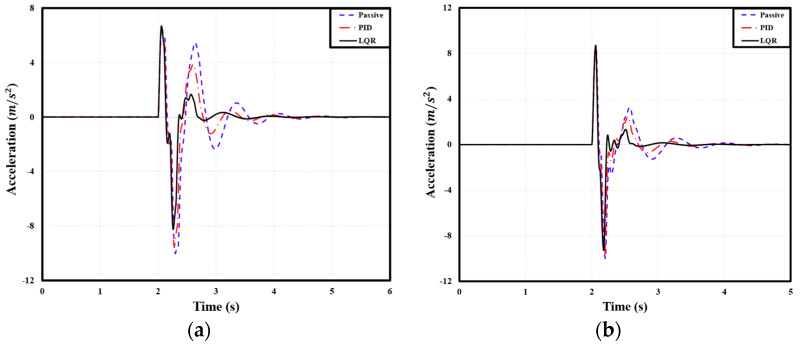
Front−axle acceleration of cargo PBV under different speed bump dimensions: (**a**) width: 3.6 m, height: 0.1 m; (**b**) width: 2.0 m, height: 0.075 m.

**Figure 21:**
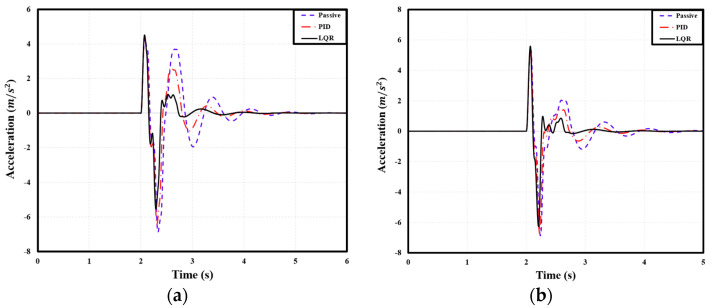
Front−seat acceleration of passenger PBV under different speed bump dimensions: (**a**) width: 3.6 m, height: 0.1 m; (**b**) width: 2.0 m, height: 0.075 m.

**Figure 22:**
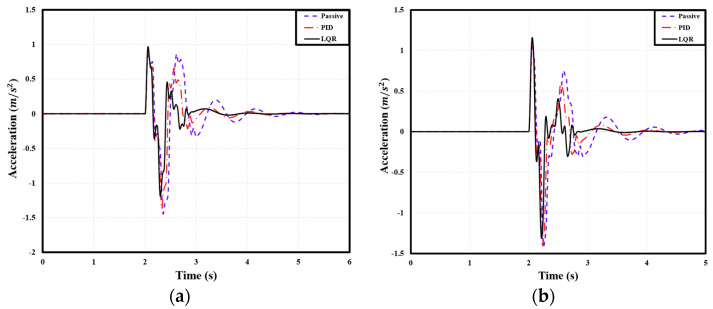
Acceleration of bed service PBV under different speed bump dimensions: (**a**) width: 3.6 m, height: 0.1 m; (**b**) width: 2.0 m, height: 0.075 m.

The peak-to-peak acceleration of the PBV was measured when it passed over two speed bumps with the following dimensions: 3.6 m width, 0.1 m height; 2.0 m width, 0.075 m height. For the cargo-transport PBV, compared with the passive suspension, the PID and LQR controllers reduced the peak-to-peak acceleration by 2.31% and 6.85%, respectively, on average. Table 10 lists the corresponding peak-to-peak acceleration values. Similarly, for the passenger-transport PBV, the PID and LQR controllers reduced the peak-to-peak acceleration by 2.13% and 6.43%, respectively, on average. Specific figures can be seen in Table 11. Finally, for the bed-service PBV, the PID and LQR controllers reduced the peak-to-peak acceleration by 0.25% and 3.45%, respectively, on average. Specific figures can be seen in Table 12. See Figure 20, Figure 21 and Figure 22.

### 4.3. Comparison of Vibration Dose Value Based on User-Defined

When a PBV is utilized for cargo transportation, ensuring stability is essential for protecting the payload from damage. The required dynamic characteristics vary according to the type of load carried by the PBV. Therefore, when a target *VDV* is specified using the velocity Equation (14), the reliability and stability of the PBV must be guaranteed only when the value falls within an acceptable error range. In this study, the error range was set at +10% from the target value. If *VDV* is lower than the target value, it is considered insignificant, and the required dynamic characteristics are assumed to be achieved. In this study, the *eVDVs* corresponding to the comfort levels in Table 2 were set as the user-defined values, and user-defined comfort was considered to be in the “Fairly uncomfortable” ($a_{w}=1$) range for the cargo-transport PBV, the “A little uncomfortable” ($a_{w}=0.63$) range for the passenger-transport PBV, and the “Not uncomfortable” ($a_{w}=0.315$) range for the bed-service PBV. 

**Figure 23:**
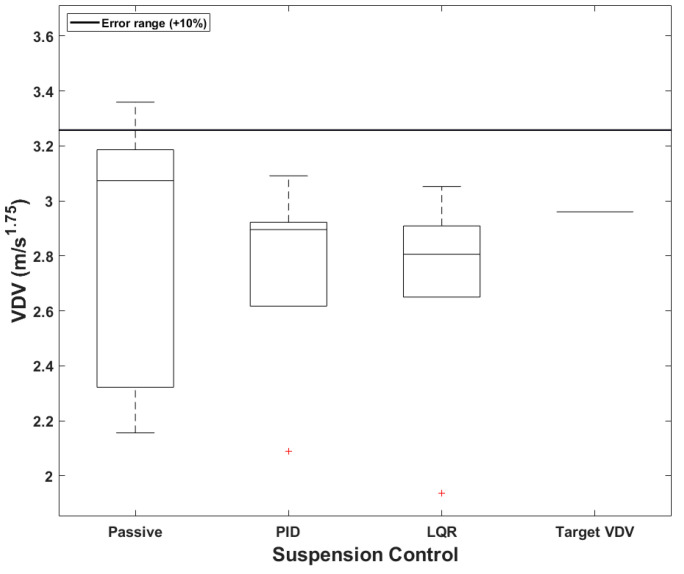
Boxplots of *VDVs* for cargo PBVs.

As seen in Figure 23, the target *VDV* for the cargo-transport PBV was 2.96, considering the requirement for fast delivery. The boxplots compare the accuracies of the suspension control methods. Evidently, all the *VDVs* fall within the acceptable error range when both the PID and LQR controllers are used. For the passive suspension system, the median value slightly exceeds the target but is still within the acceptable error range. In terms of the suspension control method, the passive, PID-based, and LQR-based systems exhibited maximum errors of 13.48%, 4.43%, and 3.11%, respectively, with respect to the target value.

**Figure 24:**
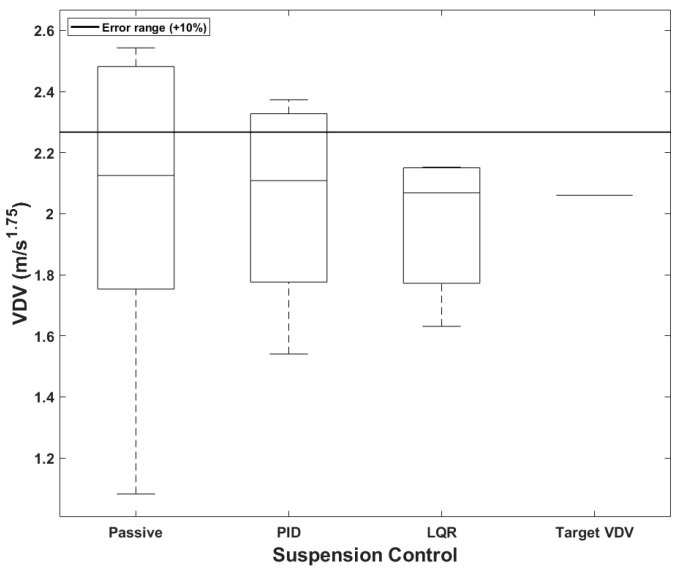
Boxplots of *VDV* for passenger PBV.

When a PBV is used for passenger transportation, a common application of mobility, it becomes essential to ensure ride comfort and alleviate motion sickness. Figure 24 presents boxplots comparing the accuracies of the different suspension control methods for the passenger-transport PBV. The target *VDV* was set at 2.06, considering the comfort requirements of passengers, see Table 13. According to Table 14, the average *VDV* was smaller than the target value for all the suspension systems, and the median value was also within the acceptable error range of 10%. Notably, *VDV* decreased progressively from the passive system to the PID-based system to the LQR-based system; this shows that the semi-active systems, particularly the LQR-based system, provided greater comfort. The maximum errors were fairly large, however, being 23.45%, 15.19%, and 4.47% for the passive, PID-based, and LQR-based systems.

**Figure 25:**
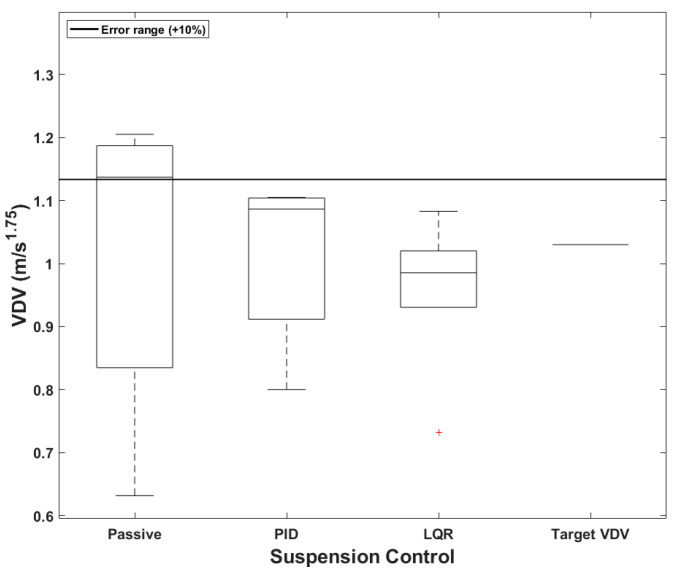
Boxplots of *VDVs* for bed-service PBVs.

For PBVs serving as ambulances or mobile hotels, it is necessary to ensure that passengers can rest comfortably on the beds. For the bed-service PBV in this study, the target *VDV* was set as 1.03, considering the requirements for emergency situations and resting on a bed. For the passive suspension, the average *VDV* was within the target value, but the median value marginally exceeded the acceptable error threshold. However, for the PID and LQR controllers, both the average and median *VDVs* were within either the target value or the acceptable error range, as seen in Table 15. In addition, the maximum errors for the passive, PID-based, and LQR-based systems were 16.99%, 7.28%, and 5.15%, respectively. See Figure 25.

## 5. Conclusions

In this study, the dynamic characteristics of three types of PBVs were analyzed considering cargo-transportation, passenger-transportation, and bed-service applications, which highlight the versatility of PBVs. The dynamic stability was evaluated as per ISO2631-1 to determine *VDVs*, and impacts at specific locations within the PBV during speed bump traversal were assessed. Furthermore, semi-active suspension control systems were employed to ensure dynamic stability when encountering speed bumps at a consistent velocity. Subsequently, a velocity-control logic was devised via multiple regression analysis to ensure stability when driving over speed bumps of various shapes.

The measured *VDVs* were compared between the front and rear axles and the front and rear seats for the cargo- and passenger-transportation scenarios, respectively; based on the higher *VDVs*, the front axle and front seat were then selected to assess the stability of the PBV when passing over speed bumps. Moreover, the dynamic instability, characterized by the *VDVs* and peak-to-peak acceleration values, was the highest for the cargo-transport PBV, followed by the passenger-transport PBV and then the bed-service PBV. Additionally, the correlation between the dynamic characteristics of the PBV and the speed bump dimensions was analyzed by measuring the *VDVs* for different speed bump widths and heights, as well as different PBV velocities. Multiple regression analysis was performed to determine the maximum velocity at which the target *VDV* could be achieved during speed bump traversal for each PBV configuration. This analysis considered factors such as the speed bump dimensions, the suspension control method, and the velocity equation based on the target *VDV*.

The velocity equation, incorporating variables such as the width and height of the speed bump and the user-defined *VDV*, was verified. For the cargo-transport PBV, compared with the passive suspension system, the PID-based and LQR-based suspension systems increased the velocity by an average of 11.01% and 24.93%, respectively, considering changes in the width of the speed bump; further, for different heights of the speed bump, the velocity increased by 10.24% and 22.13%, respectively, on average. For the passenger-transport PBV, the PID and LQR controllers increased the velocity by 23.74% and 50.74% on average, respectively, under different speed bump widths; under different speed bump heights, the PID and LQR controllers increased the velocity by 19.44% and 38.31% on average, respectively. For the bed-service PBV, the PID and LQR controllers increased the velocity by an average of 22.28% and 40.59%, respectively, with changes in the width of the speed bump; under different heights of the speed bump, the PID and LQR controllers increased the velocity by 22.28% and 40.59% on average, respectively. Further reductions are anticipated with the incorporation of deceleration speed profiles in the future.

The accuracy of the velocity equation was evaluated based on whether the target *VDVs* could be achieved by the cargo-transport, passenger-transport, and bed-service PBVs, considering an acceptable error range of 10%. For the cargo-transport PBV, the target *VDV* was set to 2.96, and the average and median *VDVs* were within the acceptable error range for all the suspension systems. In particular, with the PID and LQR controllers, the *VDV* was below the target value. Moreover, the maximum errors for the passive, PID-based, and LQR-based systems were 13.48%, 4.43%, and 3.12%, respectively. For the passenger-transport PDV, the target *VDV* was set to 2.06, and the average and median *VDVs* were found to be within the 10% threshold for all the suspension systems. The maximum errors for the passive, PID-based, and LQR-based systems were 23.45%, 15.19%, and 4.47%, respectively. For the bed-service PBV, both the mean and median *VDVs* were within the acceptable error range, and the maximum errors were 16.99%, 7.28%, and 5.15% for the passive, PID-based, and LQR-based systems, respectively. In conclusion, the maximum error rate progressively decreased from the passive system to the PID-based system to the LQR-based system for all the PBV configurations.

Based on the analysis of the dynamic characteristics of the PBVs in terms of user-defined *VDVs*, dynamic stability was achieved when the PBVs passed over speed bumps of different shapes. By utilizing the derived equations, the semi-active suspension control systems could reduce the time required for speed-bump traversal and ensure the stability of the PBV by controlling its speed.

## Figures and Tables

**Table 1:** Multiplication factors used for evaluation [7].

Vibration Direction	Multiplication Factor	Multiplication Factor Value
*x*	$k_{x}$	1
*y*	$k_{y}$	1
*z*	$k_{z}$	1.4

**Table 2:** Classification of ride comfort based on vibration [7].

Subjective Feeling	WRMS $(m/s^{2}$)
Not uncomfortable	Less than 0.315
A little uncomfortable	0.315–0.63
Fairly uncomfortable	0.5–1
Uncomfortable	0.8–1.6
Very uncomfortable	1.25–2.5
Extremely uncomfortable	Greater than 2

**Table 3:** Peak-to-peak acceleration for different PBV configuration (unit: $m/s^{2}$).

Purpose	Multiplication Factor	Multiplication Factor Value
Cargo	14.28	16.56
Passenger	12.48	14.12
Bed	4.88	5.75

**Table 4:** Velocity according to the suspension control method under different speed bump widths for cargo PBV. (unit: km/h).

Speed Bump Width (m)	Passive	PID	LQR
4.0	32.81	35.46	38.61
3.6	30.32	33.04	36.32
3.2	27.67	30.45	33.85
2.8	24.84	27.65	31.17
2.4	21.81	24.61	28.24
2.0	18.54	21.29	25

**Table 5:** Velocity according to the suspension control method under different speed bump heights for cargo PBV. (unit: km/h).

Speed Bump Height (m)	Passive	PID	LQR
0.025	53.3	53.64	55.65
0.05	45.64	46.78	49.21
0.075	37.98	39.91	42.77
0.1	30.32	33.04	36.32
0.125	22.66	26.18	29.88
0.15	15	19.31	23.44

**Table 6:** Velocity according to the suspension control method under different speed bump widths for passenger PBV (unit: km/h).

Speed Bump Width (m)	Passive	PID	LQR
4.0	27.53	30.58	33.86
3.6	24.09	27.34	30.84
3.2	20.39	23.81	27.53
2.8	16.38	19.93	23.86
2.4	12.02	15.63	19.74
2.0	7.22	10.79	15.05

**Table 7:** Velocity according to the suspension control method under different speed bump heights for passenger PBV (unit: km/h).

Speed Bump Height (m)	Passive	PID	LQR
0.025	48.53	49.02	48.53
0.05	40.38	41.79	44.19
0.075	32.24	34.57	37.52
0.1	24.09	27.34	30.84
0.125	15.94	20.11	24.17
0.15	7.80	12.89	17.50

**Table 8:** Velocity according to the suspension control method under different speed bump widths for bed PBV (unit: km/h).

Speed Bump Width (m)	Passive	PID	LQR
4.0	29.95	33.77	36.05
3.6	26.82	30.88	33.57
3.2	23.5	27.77	30.89
2.8	19.97	24.4	27.7
2.4	16.18	20.71	24.75
2.0	12.11	16.64	21.18

**Table 9:** Velocity according to the suspension control method under different speed bump heights for bed PBV (unit: km/h).

Speed Bump Height (m)	Passive	PID	LQR
0.025	52.42	52.81	52.98
0.05	43.89	45.5	46.51
0.075	35.36	38.19	40.04
0.1	26.82	30.88	33.57
0.125	18.29	23.58	27.11
0.15	9.76	16.27	20.64

**Table 10:** Peak-to-peak acceleration of cargo PBV (unit: $m/s^{2}$).

Controller	W: 3.6 m, H: 0.1 m	W: 2.0 m, H: 0.075 m
Passive	11.22	12.25
PID	10.83	12.11
LQR	10.04	11.86

**Table 11:** Peak-to-peak acceleration of passenger PBV (unit: $m/s^{2}$).

Controller	W: 3.6 m, H: 0.1 m	W: 2.0 m, H: 0.075 m
Passive	16.55	18.57
PID	16.04	18.35
LQR	14.92	18.01

**Table 12:** Peak-to-peak acceleration of bed PBV (unit: $m/s^{2}$).

Controller	W: 3.6 m, H: 0.1 m	W: 2.0 m, H: 0.075 m
Passive	2.33	2.46
PID	2.29	2.49
LQR	2.16	2.47

**Table 13:** *VDVs* of cargo PBVs with different suspension systems (unit: $m/s^{1.75}$).

Cargo	Passive	PID	LQR	Target *VDV*
Mean	2.86	2.75	2.69	2.96
Median	3.07	2.90	2.81	2.96
Maximum error	3.36	3.09	3.05	-

**Table 14:** *VDVs* of passenger PBVs with different suspension systems (unit: $m/s^{1.75}$).

Passenger	Passive	PID	LQR	Target *VDV*
Mean	2.02	2.04	1.97	2.06
Median	2.13	2.11	2.07	2.06
Maximum error	2.54	2.37	2.15	-

**Table 15:** *VDVs* of bed-service PBVs with different suspension systems (unit: $m/s^{1.75}$).

Passenger	Passive	PID	LQR	Target *VDV*
Mean	1.02	1.02	0.96	1.03
Median	1.14	1.09	0.99	1.03
Maximum error	1.21	1.11	1.08	-

## Data Availability

The data are contained within this article.

## Appendix A. PBV Modeling Differential Equations

(A1)
$$\left\{\begin{array}{c} m_{uf}{\ddot{z}}_{uf}+c_{sf}\left({\dot{z}}_{uf}-{\dot{z}}_{sf}\right)+k_{sf}\left(z_{uf}-z_{sf}\right)+k_{uf}\left(z_{uf}-z_{rf}\right)+F_{f}=0\\ m_{ur}{\ddot{z}}_{ur}+c_{sr}\left({\dot{z}}_{ur}-{\dot{z}}_{sr}\right)+k_{sr}\left(z_{ur}-z_{sr}\right)+k_{ur}\left(z_{ur}-z_{rr}\right)+F_{r}=0\\ m_{s}{\ddot{z}}_{s}+c_{sf}\left({\dot{z}}_{sf}-{\dot{z}}_{uf}\right)+k_{sf}\left(z_{sf}-z_{uf}\right)+c_{sr}\left(\dot{z_{sr}}-{\dot{z}}_{ur}\right)+k_{sr}\left(z_{sr}-z_{ur}\right)-F_{f}-F_{r}=0\\ I_{s}{\ddot{\theta }}_{s}+a\left[c_{sf}\left({\dot{z}}_{sf}-{\dot{z}}_{uf}\right)+k_{sf}\left(z_{sf}-z_{uf}\right)-F_{f}\right]-b\left[c_{sr}\left({\dot{z}}_{sr}-{\dot{z}}_{ur}\right)+k_{sr}\left(z_{sr}-z_{ur}\right)-F_{r}\right]=0\\ z_{s}=\frac{az_{sr}+bz_{sf}}{a+b}\\ \theta_{s}=\frac{z_{sf}-z_{sr}}{a+b}\\ \frac{m_{s}}{a+b}\left(a{\ddot{z}}_{sr}+b{\ddot{z}}_{sf}\right)+c_{sf}\left({\dot{z}}_{sf}-{\dot{z}}_{uf}\right)+k_{sf}\left(z_{sf}-z_{uf}\right)+c_{sr}\left({\dot{z}}_{sr}-{\dot{z}}_{ur}\right)+k_{sr}\left(z_{sr}-z_{ur}\right)-F_{f}-F_{r}=0\\ \frac{I_{s}}{a+b}\left({\ddot{z}}_{sf}+{\ddot{z}}_{sr}\right)+a[c_{sf}\left({\dot{z}}_{sf}-{\dot{z}}_{uf}\right)+k_{sf}\left(z_{sf}-z_{uf}\right)-F_{f}]-b[c_{sr}\left({\dot{z}}_{sr}-{\dot{z}}_{ur}\right)+k_{sr}\left(z_{sr}-z_{ur}\right)-F_{r}]=0 \end{array}\right.$$

(A2)
$$\left\{\begin{array}{c} m_{Pf}{\ddot{z}}_{mPf}+c_{Pf}\left({\dot{z}}_{mPf}-{\dot{z}}_{Pf}\right)+k_{Pf}\left(z_{mPf}-z_{Pf}\right)=0\\ m_{Pr}{\ddot{z}}_{mPr}+c_{Pr}\left({\dot{z}}_{mPr}-{\dot{z}}_{Pr}\right)+k_{Pr}\left(z_{mPr}-z_{pr}\right)=0 \end{array}\right.$$

(A3)
$$\left\{\begin{array}{c} m_{B}{\ddot{z}}_{B}+c_{Bf}\left({\dot{z}}_{mBf}-{\dot{z}}_{Bf}\right)+k_{Bf}\left(z_{mBf}-z_{Bf}\right)+c_{Br}\left({\dot{z}}_{mBr}-{\dot{z}}_{Br}\right)+k_{Br}\left(z_{mBr}-z_{Br}\right)=0\;\\ I_{B}{\ddot{\theta }}_{B}+e\left[c_{Bf}\left({\dot{z}}_{mBf}-{\dot{z}}_{Bf}\right)+k_{Bf}\left(z_{mBf}-z_{Bf}\right)\right]-f\left[c_{Br}\left({\dot{z}}_{mBr}-{\dot{z}}_{Br}\right)+k_{Br}\left(z_{mBr}-z_{Br}\right)\right]=0 \end{array}\right.$$

## Appendix B. PBV Model Parameters

**Table A1:** Half-car parameters.

Variables	Value	Variables	Value
$m_{s}$ (kg)	1140	$k_{sf}$ (N/m)	38,404
$I_{s}$ (kgm^2^)	3262.3	$k_{sr}$ (N/m)	38,404
$m_{uf}$ (kg)	57.55	$c_{sf}$ (Ns/m)	2693
$m_{ur}$ (kg)	53.95	$c_{sr}$ (Ns/m)	2693
$k_{uf}$ (N/m)	200,000	a (m)	1.85
$k_{ur}$ (N/m)	200,000	b (m)	1.85

**Table A2:** Seat parameters.

Variables	Value	Variables	Value
$m_{Pf}$ (kg)	8	$k_{Pf}$ (N/m)	13,530
$m_{Pr}$ (kg)	8	$k_{Pr}$ (N/m)	13,530
$c_{Pf}\;$(Ns/m)	605	c (m)	1.234
$c_{pr}$ (Ns/m)	605	d (m)	1.234

**Table A3:** Bed parameters [17].

Variables	Value	Variables	Value
$m_{B}$ (kg)	5	$k_{Bf}$ (N/m)	21,360
$I_{B}$ (kgm^2^)	1.6	$k_{Br}$ (N/m)	21,360
$c_{Bf}\;$(Ns/m)	279	e (m)	0.8
$c_{Br}$ (Ns/m)	279	f (m)	0.8

## Appendix C. Controller Gains in PID and LQR Control

**Table A4:** PID gain value.

Suspension Position	$K_{P}$	$K_{I}$	$K_{D}$
Front	1100	12,000	1300
Rear	1000	1100	1150

(A4)
$$Q_{f}=\left[\begin{array}{cccccccc} 900 & 0 & 0 & 0 & 0 & 0 & 0 & 0\\ 0 & 100 & 0 & 0 & 0 & 0 & 0 & 0\\ 0 & 0 & 300 & 0 & 0 & 0 & 0 & 0\\ 0 & 0 & 0 & 100 & 0 & 0 & 0 & 0\\ 0 & 0 & 0 & 0 & 100 & 0 & 0 & 0\\ 0 & 0 & 0 & 0 & 0 & 100 & 0 & 0\\ 0 & 0 & 0 & 0 & 0 & 0 & 100 & 0\\ 0 & 0 & 0 & 0 & 0 & 0 & 0 & 100 \end{array}\right]$$

(A5)
$$R_{f}=0.00001$$

(A6)
$$Q_{r}=\left[\begin{array}{cccccccc} 200 & 0 & 0 & 0 & 0 & 0 & 0 & 0\\ 0 & 100 & 0 & 0 & 0 & 0 & 0 & 0\\ 0 & 0 & 800 & 0 & 0 & 0 & 0 & 0\\ 0 & 0 & 0 & 100 & 0 & 0 & 0 & 0\\ 0 & 0 & 0 & 0 & 100 & 0 & 0 & 0\\ 0 & 0 & 0 & 0 & 0 & 100 & 0 & 0\\ 0 & 0 & 0 & 0 & 0 & 0 & 100 & 0\\ 0 & 0 & 0 & 0 & 0 & 0 & 0 & 100 \end{array}\right]$$

(A7)
$$R_{r}=0.00001$$

## Appendix D. Coefficient of Determination for Different PBV Configurations

**Table A5:** Coefficient of determination for different PBV configurations.

Configuration	Controller	$R^{2}$
Cargo	Passive	0.943
	PID	0.931
	LQR	0.920
Passenger	Passive	0.899
	PID	0.900
	LQR	0.895
Bed	Passive	0.892
	PID	0.902
	LQR	0.912
